# TP63 links chromatin remodeling and enhancer reprogramming to epidermal differentiation and squamous cell carcinoma development

**DOI:** 10.1007/s00018-020-03539-2

**Published:** 2020-05-23

**Authors:** Mei Yi, Yixin Tan, Li Wang, Jing Cai, Xiaoling Li, Zhaoyang Zeng, Wei Xiong, Guiyuan Li, Xiayu Li, Pingqing Tan, Bo Xiang

**Affiliations:** 1 NHC Key Laboratory of Carcinogenesis, Hunan Provincial Cancer Hospital and the Affiliated Cancer Hospital of Xiangya School of Medicine, Central South University, Changsha, 410013 Hunan China; 2 Department of Dermatology, Xiangya Hospital, Central South University, Changsha, 410008 Hunan China; 3 Hunan Key Laboratory of Nonresolving Inflammation and Cancer, The Third Xiangya Hospital, Central South University, Changsha, 410013 Hunan China; 4 The Key Laboratory of Carcinogenesis and Cancer Invasion of the Chinese Ministry of Education, Cancer Research Institute and School of Basic Medical Sciences, Central South University, Changsha, 410078 Hunan China; 5 Department of Dermatology, The Second Xiangya Hospital, The Central South University, Changsha, 410011 Hunan China; 6 Department of Thoracic Surgery, The Second Xiangya Hospital, Central South University, Changsha, 410011 Hunan China; 7 Department of Head and Neck Surgery, Hunan Provincial Cancer Hospital and Cancer Hospital Affiliated to Xiangya Medical School, Central South University, Changsha, 410013 Hunan China

**Keywords:** Epigenetic reprogramming, Oncogene addiction, SWI/SNF complex, Histone modification, Basal cell, Ubiquitin–proteasome system

## Abstract

Squamous cell carcinoma (SCC) is an aggressive malignancy that can originate from various organs. TP63 is a master regulator that plays an essential role in epidermal differentiation. It is also a lineage-dependent oncogene in SCC. ΔNp63α is the prominent isoform of TP63 expressed in epidermal cells and SCC, and overexpression promotes SCC development through a variety of mechanisms. Recently, ΔNp63α was highlighted to act as an epidermal-specific pioneer factor that binds closed chromatin and enhances chromatin accessibility at epidermal enhancers. ΔNp63α coordinates chromatin-remodeling enzymes to orchestrate the tissue-specific enhancer landscape and three-dimensional high-order architecture of chromatin. Moreover, ΔNp63α establishes squamous-like enhancer landscapes to drive oncogenic target expression during SCC development. Importantly, ΔNp63α acts as an upstream regulator of super enhancers to activate a number of oncogenic transcripts linked to poor prognosis in SCC. Mechanistically, ΔNp63α activates genes transcription through physically interacting with a number of epigenetic modulators to establish enhancers and enhance chromatin accessibility. In contrast, ΔNp63α also represses gene transcription via interacting with repressive epigenetic regulators. ΔNp63α expression is regulated at multiple levels, including transcriptional, post-transcriptional, and post-translational levels. In this review, we summarize recent advances of p63 in epigenomic and transcriptional control, as well as the mechanistic regulation of p63.

## Introduction

Squamous cell carcinomas (SCCs) are a series of aggressive malignancies from various tissue origins, including the skin, head and neck, lung, and esophagus [1, 2]. Squamous subtype cancers can also be identified in subsets of pancreatic [3–5], urothelial [6], and prostate cancer [7]. SCCs from various organs share some similar features, including their genomic landscapes, morphological characteristics, and molecular alterations [2, 8]. Human papillomavirus infection and consumption of alcohol and tobacco are defined etiology factors for SCCs [9]. To date, SCCs are highly aggressive and current therapeutics have limited effect, and do not provide a satisfactory clinical outcome [2].

**Fig. 1:**
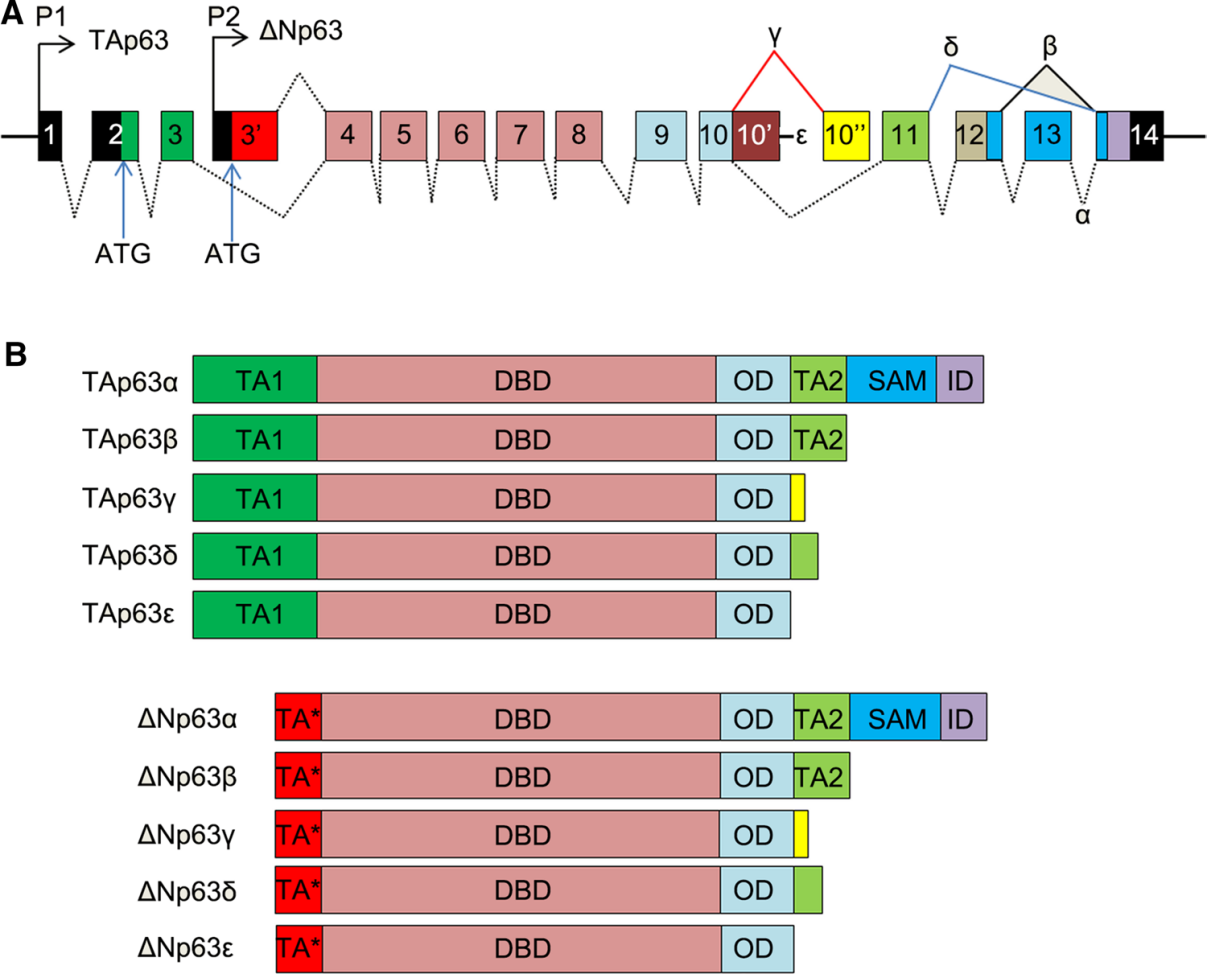
The p63 gene and protein structures. **a** Genomic structure of human TP63 gene. Alternative promoters (P1 and P2) are indicated. Alternative splicing events at the COOH terminus generate variants α, β, and γ. Exons skipping or premature transcription termination produces variants δ and ε, respectively. **b** Schematic diagrams of p63 protein isoforms structures. *TA1* transactivating domain, TA* truncated transactivating domain of ΔN isoforms, *DBD* DNA-binding domain, *OD* oligomerization domain, *TA2* secondary transactivating domain, *SAM* sterile alpha motif, *ID* inhibitory domain

Lineage dependency occurs where cancer cells depend on survival and self-renewal mechanisms co-opted from the original healthy tissues from where they arose [10]. Transcription factor (TF) TP63 is a p53 family member which plays a crucial role in epidermal development. Alternative usage of different promoters of TP63 gene results in two major isoforms, TAp63 and NH2-terminal truncated ΔNp63, which lacks the canonical transactivating domain (TA1) (Fig. 1a) [11]. However, it is proposed that ∆Np63 has an intrinsic transcription transactivation ability, which is conferred by a second transactivation domain (TA2) located between exons 11 and 12 [12]. Alternative splicing at the COOH terminal further generates variants of each isoform (α, β, γ) [11]. The δ variant is generated by the skipping of exons 12 and 13, whereas the ε variant arises from a premature transcriptional termination in intron 10 [13] (Fig. 1b). All p63 isoforms contain a central DNA-binding domain (DBD) and an oligomerization domain (OD). The α proteins are the longest isoforms, containing a COOH-terminal sterile alpha motif (SAM) domain, which mediates protein–protein interaction. SAM domain is followed by an inhibitory domain (ID), which auto-inhibits the transcriptional activity of the TA1 domain [14]. The β isoforms contain the TA2 domain, but lack both SAM and ID domains. The γ variants contain an OD domain, followed by a unique sequence derived from intron 10 (Fig. 1b) [13].

**Fig. 2:**
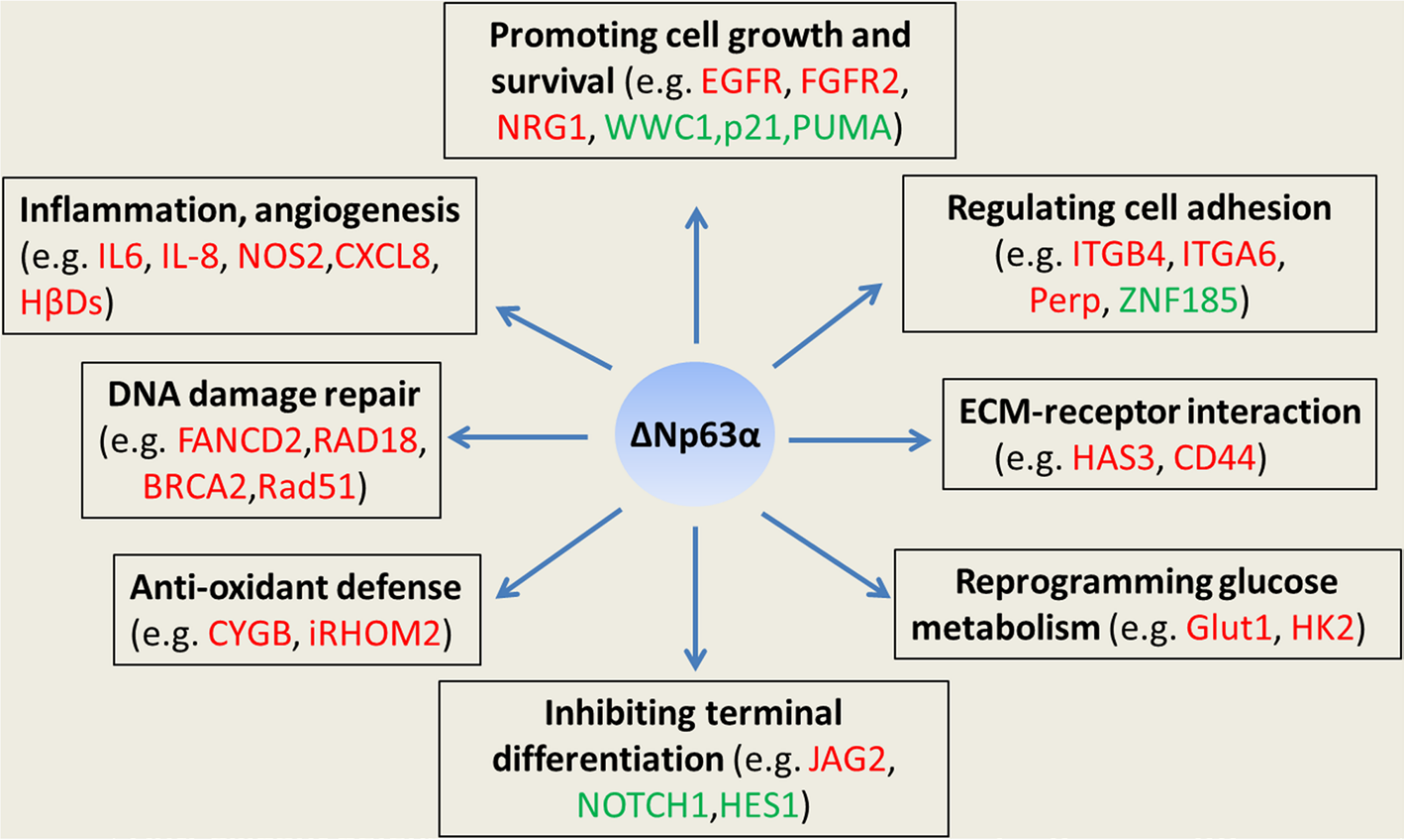
Diverse functions of ΔNp63α in SCCs. ΔNp63α promotes the development of malignant features of SCC through multiple mechanisms, including stimulation of cell growth and survival, inhibition of terminal differentiation, reprogramming of glucose metabolism and maintaining anti-oxidative homeostasis, promotion of DNA damage repair and triggering of inflammation and angiogenesis. ΔNp63α is also understood to regulate cell adhesion and remodeling of the ECM in tumor microenvironments. Representative target genes of ΔNp63α in SCC are shown. Red or green represents positively or negatively regulated target gene

The TAp63 and ∆Np63 isoforms regulate distinct target gene sets and often exert opposing regulatory functions [15, 16]. Heterozygosity of p63 prevents spontaneous and chemical-induced SCC formation [17, 18], indicating that TP63 acts as a lineage-survival oncogene in SCCs. ΔNp63α is the prominent isoform of the TP63 gene expressed in keratinocytes and basal cells of diverse epithelia [19–21] and various SCCs [2]. ΔNp63α promotes SCC development through regulating different target genes, including cell growth and proliferation [22, 23], extracellular matrix (ECM)–receptor interaction [24], cell adhesion [25–27], glucose metabolism [28, 29], anti-oxidant defense [30, 31], DNA damage repair [32, 33], terminal differentiation [34, 35], and inflammation and angiogenesis [36–41] (Fig. 2). Sustained expression of ∆Np63α tends to promote the well-differentiated SCC phenotype and restricts epithelial–mesenchymal transition induced by TGF-β [42]. However, both suppressive [43–45] and promotive roles [46] of ∆Np63α in cell invasion and metastasis have been demonstrated in various cell contexts, suggesting a cell context-specific role in cancer metastasis. Growing evidence demonstrates that ΔNp63 exerts both a suppressive and promotive role on target genes expression [47–49]. Recent studies established the master regulator role of p63 in controlling chromatin accessibility and enhancer reprogramming in keratinocytes [50, 51]. Various epigenetic modulators, including chromatin-remodeling complexes and epigenetic enzymes, are implicated in p63-mediated epigenomic reprogramming during keratinocyte differentiation and SCC development [52]. In this review, we focus on the role and mechanisms of p63 in chromatin remodeling and enhancer reprogramming during epidermal differentiation and SCC development.

## TP63 in epidermal commitment and differentiation

TP63 plays essential roles in lineage commitment during epidermal development through a variety of epigenetic mechanisms (Table 1). Mutations of TP63 are associated with various developmental disorders, including ectrodactyly–ectodermal dysplasia–cleftlip/palate (EEC) syndrome [53], ankyloblepharon–ectodermal defects–cleft lip/palate (AEC) syndrome and split-hand/foot malformation-IV syndrome. Ablation of all p63 isoforms in mice results in the absence of stratified epithelia and their derivatives [54–56], and specific ablation of ΔNp63 isoform leads to severe developmental anomalies similar to that existing in p63-null mice. These abnormalities include truncated forelimbs and the absence of hind limbs [57], indicating that the TP63 gene is required for skin and epithelial development. However, both p63- and ΔNp63-deficient epidermis expresses the terminal differentiation markers loricrin, filaggrin, and involucrin [54–56], although there is a premature expression pattern of terminal differentiation markers at E15.5 in ΔNp63 isoform-specifically ablated mice [57]. This suggests that TP63 or ΔNp63 is dispensable for epidermal lineage commitment. Intriguingly, introducing p63 mutants from EEC patients into induced pluripotent stem cells (iPSCs) dramatically impairs the induction of epidermal marker K14 in an epidermal commitment model [58] induced by retinoic acid (RA) and bone morphogenetic protein 4 (BMP4) [59], arguing that TP63 is essential for epidermal commitment. These opposite interpretations may indicate that other lineage-determining factors could compensate for p63 function in a p63-null context, whereas mutant p63 in iPSCs abrogates the function of the normal p63. Although p63 is dispensable to drive human embryonic stem cells (hESCs) to differentiate to surface ectoderm progenitor cells, it is required for further differentiation toward functional keratinocytes upon RA/BMP4 treatment [60]. It has been shown that genes expressed at an early differentiation stage are not under the control of p63. The p63 protein predominantly regulates genes during the specification switch from the multipotent state to the epidermal fate [59]. Thus, these studies suggest that p63 plays a prominent role in maturation, rather than the initiation stage of skin differentiation triggered by inductive morphogens (Table 1).

**Table 1:** Function of p63 in keratinocyte differentiation

Biological processes	p63 functions	References
Epidermal commitment	p63 plays a prominent role in maturation, rather than the initiation stage	[59, 60]
Chromatin accessibility	p63 acts as epidermal pioneer factor to open chromatin architecture	[65–67]
Enhancer reprogramming	p63 establishes keratinocyte-specific enhancer landscape	[64, 66, 77]
Nonepidermal lineage commitment	p63 represses neural genes enhancers at early stage of embryonic development	[79]

## TP63 acts as an epidermal pioneer factor to open chromatin regions

It has been speculated that TP53 family members may exert intrinsic pioneer factor activity to specify epithelial lineage-specific chromatin landscape [61], and this may be the case with p63. The p63 protein preferentially binds nucleosome-enriched regions marked with active histone modifications in epidermal keratinocytes, which are inaccessible in other lineages without p63 expression [51, 62, 63], implying that p63 is required for establishing an epidermal-specific chromatin architecture at its binding regions. The primary keratinocytes from EEC syndrome patients harboring heterozygous mutations in the p63 DNA-binding domain display a dramatic reduction of chromatin accessibility at p63-binding sites compared to normal cells [64]. By using single-cell transcriptomic and epigenomic profiling techniques, Fan et al. demonstrated that ΔNp63α binding generates open chromatin regions near or within the genes involved in epidermal fate specification of skin [65]. Furthermore, Lin-Shiao et al. used p63 mutants, lacking DNA-binding activity, to demonstrate that ΔNp63α DNA binding is required to establish keratinocyte-specific enhancers [66]. During zebrafish embryo development, p63 binding occurs prior to chromatin opening at p63-binding sites (Table 1). Remarkably, the pioneered p63-binding sites are preferentially associated with epidermal-expressing genes, as is the case of *lama5*. The chromatin accessibility of the pioneered p63-binding sites is significantly reduced in tp63^−/−^ mutants. Consequently, the levels of expression of these epidermal genes are significantly decreased in the tp63^−/−^ mutant, such as *col18a1a* [67].

**Fig. 3:**
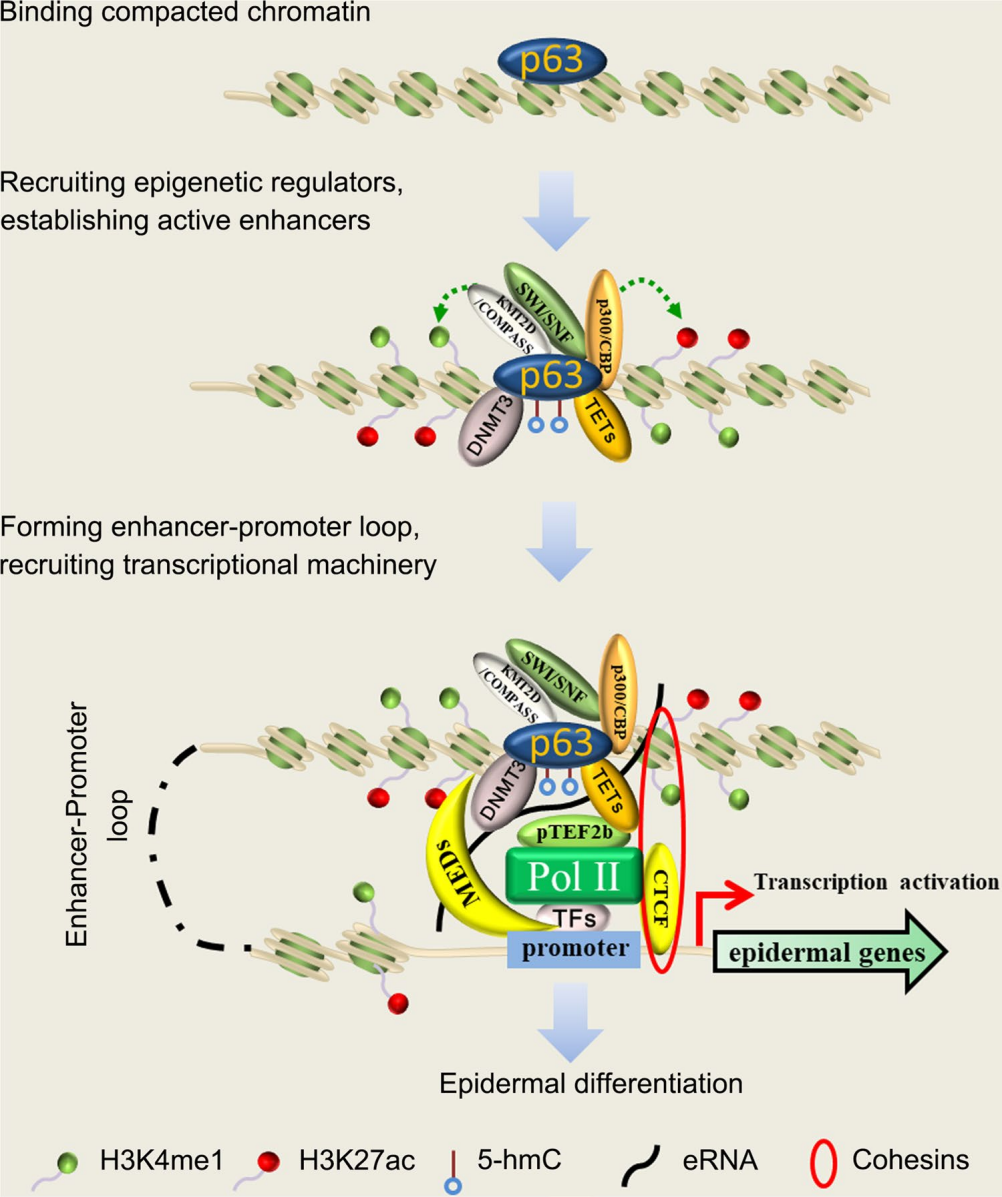
p63 acts as an epidermal pioneer factor. P63 binds to compacted chromatin regions and recruits multiple epigenetic regulators, including COMPASS complex, histone acetyltransferases p300/CBP, SNF/SWF chromatin remodeling complex, DNMT3 and TETs family members, leading to an increase in chromatin accessibility and formation of active enhancers at epidermal genes

Loss of p63 reduces the chromatin accessibility of keratinocyte-specific genes, such as KRT5. Furthermore, depletion of p63 reduces the chromatin accessibility and occupancy probability of other maturation-associated TFs, including p53, RFX, and Kruppel-like factor 4 (KLF4) [68], indicating that p63 exerts intrinsic pioneer factor activity to allow other lineage-specific TFs to bind (Fig. 3). Interestingly, ΔNp63 (−/−) epidermal cells express a number of transcripts associated with embryonic stem cells (ESCs) and induced pluripotent stem cells (iPSCs) and display compromised epithelial identity [69], suggesting that ΔNp63 exerts repressive role on genes expressed at the early differentiation stage once surface ectoderm progenitor cells enter maturation stage. Mechanistically, p63 reduces chromatin accessibility at morphogen-dependent accessible sites through promoting deposition of H3K27me3 at these sites during the maturation stage [60]. These studies consistently highlight that p63 exerts intrinsic pioneer factor activity, to open inaccessible chromatin, and it cooperates with specific TFs during keratinocyte maturation.

## TP63 establishes epidermal enhancer landscape

Enhancers are cis-regulatory elements defined by H3K4me1 modification. Enhancers can be categorized as poised or active depending on the co-occupancy of H3K27me3 or H3K27ac, respectively [70, 71]. The specific spatiotemporal regulation of developmental gene transcription is mainly determined by dynamic alterations in enhancer activity [70, 72, 73]. Active enhancers produce bidirectional enhancer RNAs (eRNAs), which can interact with various TFs and stabilize enhancer–promoter loops [74]. Remarkably, the chromatin state of enhancer regions changes more prominently than the promoter regions, as iPSCs gradually differentiate to keratinocytes [68]. Enhancers associated with genes that determine the ectoderm lineage specification, including *ITGA6*, *KRT5*, and *KRT14*, are dramatically decommissioned as epidermal stem cells (EpSCs) differentiate to keratinocytes, whereas enhancers driving the expression of epidermal differentiation genes such as *IVL* and *SPRR1A* are activated de novo, which is accompanied by a shift of Dnmt3a genomic binding from the former enhancers cluster to the latter [75].

Chromatin immunoprecipitation-sequencing (ChIP-seq) has revealed that, while the majority of p63-bound regulatory elements and p63-bound open chromatin regions in epidermal keratinocytes are active enhancers, and only a small number of p63-bound regions belong to the promoter [50, 51, 63, 64, 76], suggesting a predominant p63 role in enhancer-mediated transactivation. The p63-bound enhancers are dynamically changed during epidermal commitment. It has been demonstrated that p63 and KLF4 gain accessibility to their target enhancers as surface ectoderm progenitor differentiate to mature keratinocytes [68]. Furthermore, by using an inducible transdifferentiation model, Lin-Shiao et al. demonstrated that inducible expression of ΔNp63α in fibroblasts leads to de novo H3K27ac and an increase in chromatin accessibility at p63-bound sites, as well as expression of genes important for epithelial lineage specification [66]. Co-expression of ΔNp63α and KLF4 in fibroblasts is sufficient to activate transcription of keratin 14 and convert fibroblasts into keratinocyte-like cells. Interestingly, either ΔNp63α or KLF4 alone could not induce expression of keratin 14 [66], suggesting the interaction between ΔNp63α and KLF4 is required for full epidermal commitment. EEC syndrome patients display keratinocytes harboring p63 mutations, which lack DNA-binding ability, and display a loss of enhancers at epidermal-expressing genes, indicating that ΔNp63α binding of DNA is necessary to establish the epidermal enhancer landscape and epidermal commitment [64, 77]. Furthermore, the majority of p63-bound epidermal enhancers lose their accessibility in p63 knockout ESC during in vitro differentiation [59]. Taken together, these studies indicate that the formation and maintenance of epidermal enhancers at p63-binding sites are heavily dependent on p63 expression and its binding to DNA (Table 1).

## TP63 represses nonepithelial genes

It has been demonstrated that embryonic epithelia from p63-null mice display compromised epithelial fate, concomitant with the activation of mesodermal-specific genes [78], suggesting that p63 represses the nonepithelial transcriptional program. Introducing p63 mutants from EEC patients into iPSCs impairs the acquisition of epithelial identity, but also activates mesodermal genes expression. In contrast, inhibition of mesodermal induction by suramin enhances epidermal differentiation of iPSCs carrying the p63 mutation [59]. Cbx4, a component of polycomb repressive complex 1 (PRC1), is a target of p63 and contributes to repression of nonepithelial genes during epidermal differentiation [79]. In the early stages of embryonic development, p63 prevents Sox3 binding to pre-established neural genes enhancers and represses neural gene expression, demonstrating that p63 restricts neural lineage determination during the early stages of embryonic development [67] (Table 1). Thus, p63 safeguards epithelial lineage commitment, not only directly activating epidermal genes, but also indirectly repressing nonepithelial genes.

## TP63 establishes a squamous subtype-specific enhancer landscape in SCCs

Chromatin states are closely associated with tumorigenesis and tumor phenotypes in SCCs [42]. There are dramatic epigenetic changes between SCC and its healthy counterpart, with gain or loss of H3K27ac occupancy at various chromatin regions [80]. As a lineage-survival oncogene in SCCs, ΔNp63α is exploited by tumor cells to establish squamous-specific chromatin architecture, which favors the development of SCC [81, 82]. ΔNp63α is highly expressed and predicts unfavorable overall prognosis in squamous subtype of pancreatic cancer (PC) or pancreatic ductal adenocarcinoma (PDA) [81, 83]. ΔNp63α selectively exerts an oncogenic role in squamous-like PC [81, 82]. H3K27ac profiling demonstrates that ΔNp63α-expressing PDAs display a distinct enhancer landscape resembling the squamous cell lineage, whereas ΔNp63α occupancy in squamous-like PC cells mainly distributes on active enhancers marked with H3K27ac and open chromatin regions, rather than transcriptional start sites. Loss of ΔNp63α in squamous-like BxPC3 PDA cells selectively reduces H3K27ac at p63-bound squamous elements, whereas it exerts no effect on H3K27ac at the control regions [81]. Remarkably, a large set of ΔNp63α-occupied regions are characterized as super enhancers (SE) [82], which are believed to drive oncogene expression in multiple cancers [84, 85]. H3K27ac occupancy at SEs associated with *FAT2* and *NECTIN1* is significantly reduced upon depletion of ΔNp63α in squamous-like PC cells, as well as downregulation of *FAT2* and *NECTIN1* [82]. This indicates that SE regions are associated with the squamous subtype and display a high dependence on ΔNp63α. Nasopharyngeal carcinoma (NPC) is a unique subtype of head and neck cancer [86, 87]. ΔNp63α is the primary isoform of TP63 expressed in NPC cells [21, 88, 89]. Our works recognized that ΔNp63α is enriched in SEs associated with oncogenes in NPC, including EGFR, CD44, etc.[90, 91]. Overexpression of ΔNp63α led to preferential expression of basal cell-specific proteins, including basal-type keratins, in NPC, through establishing basal-specific SEs [90]. In addition to protein-coding genes, ΔNp63α also activates transcription of a number of long non-coding RNAs, including CCAT1 [92] and LINC01503 [93], through establishment of squamous-specific SEs in SCCs. Consequently, overexpression of CCAT1 or LINC01503 promotes progression of SCCs [92–94]. New findings suggest that phase-separated condensates of TFs and coactivators play a role in the formation of SE [95, 96]. It has been demonstrated that activation domains of diverse TFs facilitate formation of phase-separated condensates with coactivators to drive gene transcription [97]. SEs are sensitive to chemical disruption of phase separation by 1,6-hexanediol (1,6-HD) [96, 98]. It is proposed that phase separation could be a target of next-generation drugs for chromatin biology [99]. Thus, enrichment of ΔNp63α in SEs associated with oncogenes provides an opportunity to target SCC cells by disrupting phase separation.

It has been demonstrated that ΔNp63α physically interacts with SOX2 in SCC cells [100], which is a lineage-survival oncogene frequently amplified in SCC from various organ sites [101–104]. Notably, ChIP-seq analysis revealed that SOX2 and p63 co-ordinate to occupy a large set of distal enhancers in SCC cells, but co-occupancy of SOX2 and p63 is rare in embryonic stem (ES) cells, which is in line with the absence of p63 in ES cells. Depletion p63 attenuates SOX2 enrichment at the enhancer of target genes, such as ETV4, which is an oncogene co-regulated by these two proteins. However, loss of p63 exerts little effect on other SOX2-occupied regions without p63 binding [100]. Thus, this study indicates that p63 might act as a pioneer factor to establish open chromatin architecture to facilitate access by other lineage-survival factors, such as SOX2, to bind and activate target gene transcription during SCC development. Recently, it has been demonstrated that p63 cooperates with SOX2 to activate intronic enhancer cluster of GLUT1 (SLC2A1), which in turn facilitates GLUT1-mediated glucose influx and generation of NADPH and GSH in SCCs [28]. The p63 protein also binds to the enhancer region of ZNF185 and promotes its expression in keratinocyte during epithelial differentiation. However, ZNF185 is downregulated in HNSCC, esophageal and cervical SCC even though p63 is commonly amplified in these cancers [27]. It is not clear why ZNF185 is decreased in SCC, and may be due to loss of function mutations on coactivators bound to p63, which results in a decrease of enhancer activity of ZNF185 in SCC cells. In contrast, the upstream enhancer of FANCD2 is inactive in primary keratinocytes, but aberrantly activated by ΔNp63α in SCC [32].

## Mechanisms of ΔNp63α mediated chromatin remodeling and enhancer formation

There are multiple epigenetic modulators implicated in regulating enhancer and spatiotemporal transcription of lineage-specific genes, including enzymatic regulators histone methylases, histone acetyltransferases, histone deacetylases, polycomb repressive complex (PRC), DNA methylase and a number of chromatin remodelers. The p63 activates enhancers and increases chromatin accessibility through interaction with these various epigenetic modulators, which will be discussed in the section below.

## Complex of proteins associated with set1-like complex (COMPASS)

COMPASS is a multiple-protein complex, which exerts histone H3K4 methylase activity. In mammalian cells, incorporation of H3K4me1 at enhancers is mainly accomplished by histone lysine methyltransferase 2C (KMT2C/MLL3) and 2D (KMT2D/MLL4) COMPASS-related complexes [105]. UTX is a putative demethylase within the MLL3/4 COMPASS-like complex, which erases H3K27me2/3 and facilitates CBP/p300-mediated H3K27ac deposition at poised enhancers [106]. It has been demonstrated that KMT2D interacts with p63 on chromatin and occupies p63-bound enhancers (Fig. 3). Specifically, KMT2D physically interacts with the α, β, and γ isoforms of ΔNp63, indicating that the C-terminal SAM or the TA domain is not necessary for this interaction. Depletion of KMT2D in keratinocytes reduces H3K4me1 and H3K27ac at p63-binding enhancers, as well as repressing p63 target genes. Bioinformatic analysis demonstrates that KMT2D-dependent p63 target genes that lose enhancer histone modifications are enriched for development and differentiation [107]. Functionally, keratinocytes with a KMT2D deficiency display premature and highly disorganized morphologies [107]. Although a high level of protein expression was achieved, overexpression of p63 in KMT2D-depleted keratinocytes was unable to recapitulate differentiation gene expression patterns [107]. This study indicates that recruitment of KMT2D by p63 is a prerequisite to establish the active enhancer of its target genes. Given that KMT2D is broadly implicated in enhancer formation and transcription regulation, it is proposed that KMT2D might affect epidermal differentiation through multiple TFs, rather than solely through p63. KMT2D is frequently mutated in human cancers, including SCC from various organ origins [108–110], but it is not clear whether p63-KMT2D interaction occurs in cancer cells, or whether this might be the case for other histone lysine methyltransferase members.

## Histone acetyltransferases p300

Deposition of H3K27ac at the H3K4me1-defined enhancer is proposed to be catalyzed by the cAMP response element-binding protein (CBP) or p300 histone acetyltransferases [111]. Transcriptional co-activator p300 interacts with the N-terminal domain of p63γ and stimulates p63γ transcriptional activity in an acetylase-dependent manner [112]. The p300 also physically interacts with ΔNp63α and catalyzes its acetylation on lysine 193 (K193), leading to enhanced ΔNp63α protein stability [113]. A new finding demonstrated that the C-terminal domain of p63α interacts with p300 to activate β-catenin [114]. Inhibition of p300 activity suppresses HNSCC tumor growth [115, 116]. Thus, we propose that ΔNp63α might recruit p300/CBP to its bound regions to deposit H3K27ac at H3K4me1-defined enhancer (Fig. 3). Paradoxically, loss of function mutations of CBP/p300 are frequent in cutaneous SCC, suggesting a tumor-suppressor role. Loss of CBP/p300 in mouse keratinocytes exacerbates Hras^S35^-induced skin tumorigenesis [117]. Thus, it cannot be ruled out that other histone acetyltransferase members mediate ΔNp63α-dependent enhancer formation in SCC; this may be the case with general control nonrepressed protein 5 (GCN5), which is a histone acetyltransferase upregulated in ESCC [118]. It has been demonstrated that GCN5 cooperates with chromatin remodeler Switch/sucrose nonfermentable (SWI/SNF) to activate gene transcription [119, 120]. Furthermore, recruitment of SWI/SNF by GCN5 plays an essential role in the DNA damage response to double-strand breaks [121]. This suggests that the interaction between histone acetyltransferase members and the SWF/SNF complex may contribute to epigenomic reprogramming in SCC development.

## SWI/SNF/Baf complex

The mammalian SWI/SNF complex is an ATP-dependent chromatin remodeling and histone acetylation complex highly expressed in pluripotent stem cells [122]. The mSWI/SNF or BAF complex contains one catalytic subunit (Brg1 or Brm) and 14 regulatory subunits [123, 124]. It has been shown that SWI/SNF complexes interact with p300 to modulate H3K27ac and are essential for the maintenance of lineage-specific enhancers [125]. The BAF complex is required to maintain epidermal cell type-specific open chromatin sites. BAF complex and p63 protein in keratinocyte are physically close to each other (Fig. 3). A large number of open chromatin regions in epidermal cells are co-occupied by BAF and p63. BAF depletion selectively reduces the chromatin accessibility at p63 motif regions, but not at motif regions of other factors, such as CTCF and KLF4, suggesting that BAF is required for maintaining the open status of p63-bound chromatin regions. Loss of p63 selectively attenuates BAF binding to p63 regulatory elements, without affecting BAF binding to the motif of CTCF and KLF4 [51]. These data strongly suggest that ΔNp63α recruits the BAF complex to p63-bound chromatin regions, which in turn establishes an open chromatin landscape in epidermal cells. Brg1 is required for nuclear internalization of the epidermal differentiation complex (EDC) locus, which is a ~ 3.1 Mb gene-rich region of mouse chromosome 3 encoding multiple genes essential for epidermal stratification and barrier formation [126]. During development of the epidermis, EDC is relocated from the nuclear membrane toward the nuclear interior, accompanied by a developmentally linked increase in the transcriptional activity of genes within the EDC locus. It has been shown that p63 directly activates Brg1 transcription. Nuclear internalization of the EDC locus is markedly impaired in the skin epithelium of p63^−/−^ mice and is accompanied by insufficient expression of EDC genes in epidermal progenitor cells [126]. The specialized adenine and thymine-rich binding protein 1 (Satb1) is another direct target of p63 implicated in remodeling chromatin architecture of the EDC locus. Satb1 ablation, strikingly, expands the length of the whole EDC locus and its central domain, accompanied by insufficient expression of epidermis-specific genes and epidermal morphology alterations similar to p63 deficiency in skin [127]. Thus, these studies uncover an essential role of p63 in nuclear positioning of EDC locus and regulating higher-order chromatin structure of EDC through its direct target Brg1 [126] and Satb1 [127], respectively.

## DNA methyltransferase 3A (Dnmt3a) and methylcytosine dioxygenase TET2

In addition to histone modifications, the status of DNA methylation also influences the activity of enhancers. Early studies suggest that patterns of 5-methylcytosine can mark enhancers. It is widely appreciated that lineage-specific enhancer regions are usually hypomethylated [128–131]. It has been demonstrated that genomic 5-hydroxymethylcytosine (5-hmC) signals are distinctly enriched at enhancers marked with H3K4me1 in human and mouse embryonic stem cells [132, 133], suggesting a potential role in the regulation of enhancer formation. 5-hmC is enriched at the poised enhancers in mouse embryonic stem cells, rather than active enhancers with H3K27ac marks [134]. However, another study indicates that eRNA-producing enhancers in mouse embryonic stem cells are usually marked with H3K27ac, decreased DNA methylation, and are enriched in the DNA hydroxylase Tet1 [135]. In pancreatic cancer cells, 5-hmC-enriched loci specifically overlap with H3K4me1 marked enhancers and open regions of chromatin. Gain of 5-hmC is correlated with upregulation of the cognate transcripts known to be important to cancer development [136]. DNA methylation in the human genome is catalyzed by DNA methyltransferase (DNMT), including Dnmt1, Dnmt3a, and Dnmt3b. Dnmt3a and Dnmt3b catalyze de novo methylation, whereas Dnmt1 is responsible for the maintenance of genome methylation patterns [137]. Three Tet proteins catalyze DNA hydroxymethylation [138–140] and are required for eRNA production [135]. It has been demonstrated that Dnmt3a and Dnmt3b bind to active enhancers in a histone H3K36me3-dependent manner in human EpSCs. Strikingly, Dnmt3a predominantly locates at the center of its target enhancers, whereas Dnmt3b peaks broadly distribute at the enhancer center and body. Both Dnmt3a and Dnmt3b promote enhancer activity and favor binding to SEs rather than typical enhancers. The center of enhancers bound by Dnmt3a exhibit high levels of 5-hmC. Silencing Dnmt3a reduces the levels of 5-hmC at its target enhancers, whereas silencing Tet2 specifically restores the level of DNA methylation at Dnmt3a-bound enhancers, indicating that Dnmt3a-bound enhancers are sequentially methylated by Dnmt3a and then are hydroxymethylated by Tet2 to license their active state. Remarkably, Dnmt3a, not Dnmt3b, physically interacts with p63 in EpSCs [75]. Co-occupancy by p63 was seen in approximately 50% of the Dnmt3a-bound enhancers (Fig. 3), which associate with the expression of genes implicated in keratinocyte proliferation and cellular identity specification. Notably, depletion of p63 reduces Dnmt3a localization to its target enhancers [75]. During epidermal differentiation, the genomic localization of Dnmt3a shifts from the enhancers closest to genes involved in stem cell proliferation to those that regulate genes involved in differentiation [75]. We speculate that p63 sequentially recruits Dnmt3a and Tet2 localization to the center of its target enhancers, establishing high levels of 5-hmC at those sites, which facilitates subsequent deposition of H3K4me1 and H3K27ac modifications and eRNA transcription (Fig. 3). It is worth noting that ΔNp63α binds to Dnmt3a, HDAC9, and KDM4C in cisplatin-resistant SCC-11 cells, suggesting that ΔNp63α may recruit these enzymes to shape the epigenome of SCC cells during acquisition of chemoresistance [141].

## Mechanisms underlying ΔNp63α mediated transcription repression

Not only does ΔNp63α act as a dominant negative competitor to repress p53-mediated transcription [11], but it also exerts a repressive effect on transcription through interaction with diverse epigenetic regulators, which have been discussed in detail below.

## HDAC1 and HDAC2

**Fig. 4:**
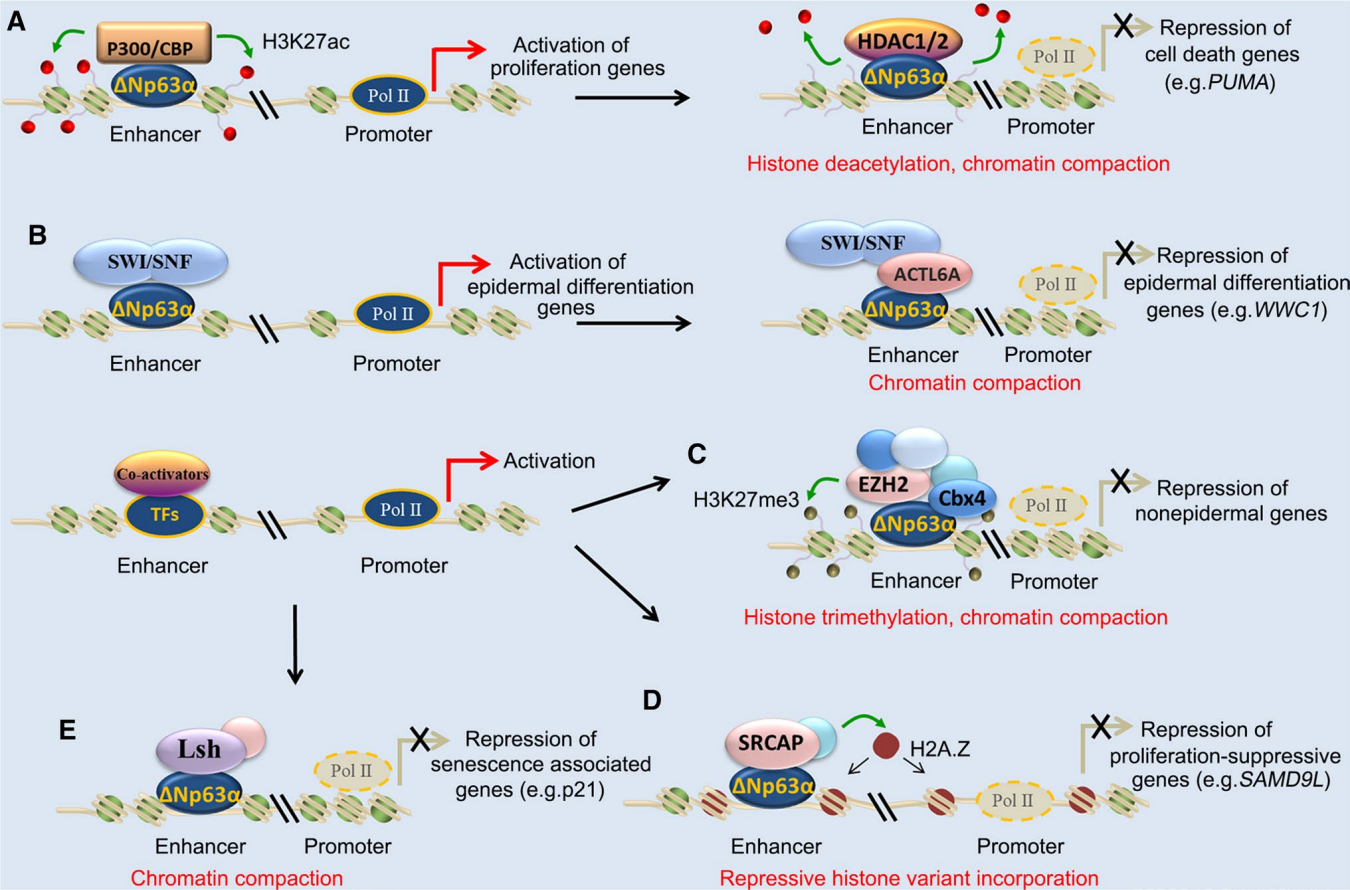
Epigenetic mechanisms underlying p63 mediated transcription repression. **a** ΔNp63α physically interacts with HDAC1/2 to remove H3K27ac at enhancer and promoter of target genes, resulting in chromatin compaction and transcriptional repression. **b** ΔNp63α normally cooperates with the SNF/SWF complex to activate gene transcription, whereas the interaction between ΔNp63α and ACTL6A prevents the SNF/SWF complex from binding to target genes, leading to repression of growth inhibitory gene WWC1. **c** ΔNp63α physically interacts with Cbx4, a component of the PRC1 complex, to repress the transcription of nonepidermal lineage genes. **d** ΔNp63α promotes incorporation of histone variant HA2.Z through recruiting SRCAP to p63-binding sites at tumor-suppressor genes. **e** ΔNp63α activates Lsh expression and cooperates with Ras to bypass OIS through repressing p21WAF1/Cip1

By using a tandem affinity purification strategy, Ramsey et al. observed that ΔNp63α interacts with histone deacetylase (HDAC) 1 and HDAC2 via its transactivation inhibitory domain in SCC cells. ΔNp63α recruits HDAC1 binding to promoter region of PUMA and represses its transcription. Treatment with the HDAC inhibitor, trichostatin A (TSA), alleviates repression of PUMA transcription by ΔNp63α and induces substantial apoptotic cell death in SCC cells. TSA treatment increases H4 acetylation levels at the p63-binding site within the PUMA promoter, whereas silencing ΔNp63α promotes deposition of H4 acetylation level at PUMA promoter and induces PUMA expression, indicating that ΔNp63α represses gene transcription through HDAC1-mediated histone deacetylation (Fig. 4a) [142]. An earlier study showed that deletion of ectodermal Hdac1 and Hdac2 leads to severe defects in hair follicle specification and epidermal proliferation and stratification, which is similar to phenotypes of p63-deficient mice [143]. Ectodermal Hdac1/2 deficiency activates the expression of targets of p63-mediated repression, rather than affecting expression of p63 positively regulated basal cell targets [143]. HDAC complexes can bind both promoter and enhancer regions of target genes [144]. While there are no studies demonstrating co-occupancy of p63 and HDAC1/2 complex on enhancers, we speculate that removal of H3K27ac by HDAC1/2 might be implicated in ΔNp63α-mediated repression of specific enhancers (Fig. 4a), since loss of ΔNp63α also results in unexpected gain of enhancer activity at RUNX1-enriched regions [77]. It has been demonstrated that the balance of EP300 and HDAC1 activities controls nucleosome eviction by the BRG1-containing SWI/SNF complex, which in turn affects transcription of DNA repair enzymes in macrophages [145]. Furthermore, recruitment of BRG1 by HDAC2 contributes to the transcriptional repression function of the SWF/SNF complex [146, 147]. Thus, it is proposed that the interaction between HDACs and the chromatin-remodeling SWF/SNF complex may be involved in ΔNp63α-mediated transcription repression.

## ACTL6A/Baf53a

ACTL6A/BAF53a is a regulatory subunit of the SWI/SNF complex implicated in embryonic stem cells pluripotency and epidermal progenitor cells state [148–150]. An early study shows Baf53 forms a distinct histone acetyltransferase complex and acts as a cofactor for c-myc in oncogenic transformation [151]. High expression of BAF53a blocks myogenic differentiation and promotes cell proliferation in rhabdomyosarcoma [152], suggesting that ACTL6A/BAF53a is implicated in cancer development. The p63 and ACTL6A proteins are rarely co-expressed in normal epidermis, but are co-amplified in a subset of HNSCC (~ 19%) [153]. ACTL6A and p63 physically interact, cooperatively controlling a transcriptional program that promotes proliferation and suppresses differentiation, in part through activation of the Hippo–YAP pathway via regulators including WWC1 (Fig. 4b). Thus, ACTL6A and p63 collaborate as oncogenic drivers in HNSCC [153]. ACTL6a suppresses epidermal differentiation of adult stem cells by preventing the SWI/SNF complex from binding to genes controlling differentiation [148]. Given that more than 20% of human malignancies carry mutations in the mSWI/SNF complex [154, 155], overexpression of ACTL6A in SCCs may severely disrupt the epidermal differentiation program controlled by the p63-mSWI/SNF complex.

## Polycomb repressive complex 1 component Cbx4

Cbx4 belongs to PRC1, which is one of the two complexes formed by polycomb chromatin-remodeling proteins that exert a repressive role on gene transcription via compacting chromatin [156]. It has been shown that Cbx4 physically interacts with p63 in thymic epithelial cells and plays an essential role in in thymic organogenesis [157]. Cbx4 is a direct target of p63 and is upregulated in keratinocytes during epithelial stratification. Ablation impairs epidermal progenitor cell proliferation and terminal differentiation [79]. Mechanistically, Cbx4 represses nonepidermal lineage gene expression via interacting with H3K27me3 that is deposited by EZH2 from the PRC1 complex and promoting ubiquitination of H2AK119 at these sites [79]. As a consequence, ubiquitination of H2AK119 is required for efficient repression of target genes by the PRC1 complex [158]. Restoration of Cbx4 partially rescues the epidermal phenotype in embryonic skin explants upon p63 ablation [79]. Thus, p63 represses the nonepidermal lineage transcription program during keratinocyte development via PRC1-mediated transcriptional repression (Fig. 4c). Cbx4 is frequently amplified and plays an oncogenic role in esophageal SCC [159], suggesting that an interaction between p63 and Cbx4 might also contribute to SCC development. A new study highlights the cooperation between mSWI/SNF and PRC1 that ensures meiotic progression in spermatocytes [160]. Furthermore, the SWI/SNF complex has been demonstrated to mediate eviction of PRC1 and PRC2 from the INK4b-ARF-INK4a locus [161]. Thus, the close interconnectivity of diverse chromatin modulators may be involved in ΔNp63α-regulated dynamics of chromatin in cancer cells.

## SRCAP chromatin regulatory complex

H2A.Z is a histone variant usually incorporated into chromatin associated with gene promoters and enhancers. SRCAP (SWR1) is the major chromatin-remodeling complex responsible for catalyzing the incorporation of H2AZ-H2B dimers into nucleosomes [162, 163]. Study revealed that ΔNp63α physically interacts with and recruits SRCAP chromatin-remodeling complex subunits, including DMAP1, RUVBL1, and RUVBL2 [164]. Recruitment of the SRCAP complex by ΔNp63α facilitates H2A.Z deposition at ΔNp63α target loci and represses its transcription (Fig. 4d). Silencing SRCAP subunits or H2A.Z specifically induces expression of ΔNp63α-repressed genes. Among those repressive target genes, SAMD9L was identified as a crucial proliferation-suppressive gene for SCC development, whose depletion alleviating proliferation arrest was caused by loss of ΔNp63α [164].

## Lymphoid-specific helicase (LSH)

Lsh is an ATPase that belongs to the SNF2/helicases family involved in chromatin remodeling [165]. Bypassing oncogene-induced senescence (OIS) is a prerequisite step for malignant transformation [166]. A pilot study found that inducible p63 deficiency in the adult epidermis leads to cellular senescence and accelerated aging [167]. It has been shown that ΔNp63α expression in primary keratinocytes is downregulated in OIS induced by oncogenic H-Ras-V12. Forced expression of wild-type ΔNp63α, rather than TAp63a or ΔNp63α^R279H^ mutant causing EEC syndrome, is sufficient to bypass OIS in Ras-expressing primary keratinocytes, indicating downregulation of ΔNp63α is required for OIS. Mechanistically, ΔNp63α cooperates with Ras to promote proliferation and survival of keratin 15-expressing stem cells. Lsh was identified as a direct target of ΔNp63α, which initiates senescence bypass, as silencing of the gene leads to a stronger senescent-like morphology [168]. Both p63 and Lsh are robustly detected in proliferating cells in DMBA-induced premalignant papillomas and HNSCC samples [168]. In addition to previously known functions regulating DNA methylation level at repeat elements and transcriptional silencing [169, 170], Lsh was recently recognized to regulate accessibility and H3 occupancy at a subset of putative enhancers associated with cellular identity, independent of DNA methylation [171, 172]. Lsh is highly expressed in various human cancers and drives cancer development [173–175]. It is proposed that Lsh may physically interact with ΔNp63α to regulate nucleosome positioning and H3 occupancy at cancer-associated genes (Fig. 4e).

## Regulation of p63 in SCC

The N6-methyladenosine (m6A) modification, which is added by methyltransferase-like 3 (METTL3), promotes the increased expression of ΔNp63α mRNA and accelerates proliferation of cutaneous SCC [176]. There are data to support the hypothesis that it does so via stabilization of the ΔNp63α mRNA transcript [177]. Along with transcriptional and post-transcriptional control, TP63 is post-translationally regulated (Table 2). Nucleoporin 62 (NUP62), a component of nuclear pore complex, interacts with ΔNp63α and promotes ΔNp63α nuclear import and ΔNp63α-dependent target gene expression. In contrast, activated ROCK1 abolishes NUP62–ΔNp63α interaction and ΔNp63α nuclear accumulates in SCC cells through phosphorylating the FG domain of NUP62 [178]. Ubiquitin–proteasome system regulated p63 protein stability is intensively studied. To date, at least three E3 ligases, including anaphase-promoting complex/cyclosome (APC/C) complex, Itch (HECT, homologous E6-AP carboxyl terminus), and WWP1, have been recognized as mediators of p63 degradation in different contexts [179]. The ΔNp63α protein is preferentially degraded during early mitosis by the APC/C complex. ΔNp63α physically associates with the 29 amino acids at the C-terminal of Cdc20, which is an important subunit for APC/C activation. Ablation of Cdc20 substantially increases the half-life of the ΔNp63α protein in proliferating keratinocytes. However, in differentiating keratinocytes, Cdh1-APC/C is required for degradation of ΔNp63α protein and maintaining terminal differentiation capacity [180]. Syntaxin binding protein 4 (Stxbp4) is a PDZ domain containing adapter protein that associates with ΔNp63α [181, 182]. It has been demonstrated that Stxbp4 prevents degradation of ΔNp63α by the Cdc20-APC/C complex and suppresses epidermis differentiation [180]. High expression of Stxbp4 protein correlates with ΔNp63α protein accumulation in both skin SCC [180] and lung SCC [182], and predicts unfavorable overall survival (OS) and progression-free survival (PFS). Itch is a Nedd4-like ubiquitin protein E3 ligase belonging to the C2-WW-HECT type E3 family, which includes the members NEDD4, WWP1, SMURF1, and SMURF2 [183, 184]. Itch associates with both TAp63α and ΔNp63α via its WW2 domain, but preferentially to the latter [185]. Co-expression of Itch together with either TAp63α or ΔNp63α leads to increase of p63 ubiquitination, whereas a catalytically inactive Itch mutant (C830A mutant) still binds to TAp63α or ΔNp63α but fails to induce its ubiquitination. The ΔNp63α protein level increases in Itch knockout (KO) primary keratinocytes, whereas reintroduction of wild-type Itch in Itch KO cells leads to reduction of endogenous ΔNp63α protein level. The conserved threonine (T538) phosphorylation of a (T/S)P motif near the PY motif of p63 stabilizes the binding of PPPPY motif with the WW2 domain of Itch, facilitating subsequent ubiquitination and degradation [185]. However, a post-phosphorylation conformation switch from *trans* to *cis* catalyzed by Pin1, a peptidyl-prolyl isomerase, prevents the binding of p63 with Itch, thus stabilizing p63 protein [186]. These studies suggested the binding of p63 with WW domain containing E3 ligases is regulated by a Pin1-mediated *cis*/*trans* conformation switch, since E3 ligases favor the *trans* conformation [187]. Stxbp4 also inhibits Itch-mediated ΔNp63α degradation. However, silencing endogenous Itch fails to stabilize ΔNp63α protein upon loss of Stxbp4 in keratinocytes, whereas silencing of the receptor for protein kinase C1 (RACK1) partially rescues ΔNp63α degradation triggered by Stxbp4 deficiency [181]. This suggests that different E3 ligases control p63 protein turnover in a context-dependent manner.

**Table 2:** Post-translational regulation of ΔNp63α protein

Regulators	Biochemical features	Biological effects and mechanisms	References
Nucleoporin 62 (NUP62)	Subunit of nuclear pore complex	NUP62 physically interacts with ΔNp63α protein and promotes ΔNp63α nuclear import and ΔNp63α-dependent target gene expression	[178]
ROCK1	Protein kinase	ROCK1 phosphorylates NUP62 and abolishes ΔNp63α–NUP62 interaction, facilitating ΔNp63α nuclear export into the cytoplasm	[178]
Cdc20-APC/C complex	E3 ligase	Cdc20 physically associates with ΔNp63α, leading to its degradation by APC/C complex in proliferating keratinocytes	[180]
Cdh1-APC/C complex	E3 ligase	Cdh1-APC/C complex promotes ubiquitination and degradation of ΔNp63α protein in differentiating keratinocytes	[180]
Itch	E3 ligase	Itch associates with ΔNp63α and induces its ubiquitination and degradation	[185]
WWP1	E3 ligase	WWP1 physically associates with both TAp63α and ΔNp63α, leading to ubiquitination and degradation of TAp63α and ΔNp63α proteins	[188]
RACK1	WD-40 repeat-containing scaffold protein	RACK1 binds ΔNp63α and promotes its degradation	[181, 191]
Stxbp4	PDZ domain containing adapter protein	Stxbp4 physically associates with ΔNp63α and prevents its degradation by the Cdc20-APC/C complex, Itch, and RACK1	[180–182]
Pin 1	Peptidyl-prolyl isomerase	Pin1 stabilizes ΔNp63α through preventing ΔNp63α-Itch association. Pin1 also inhibits ΔNp63α degradation by WWP1	[186, 189]
Stratifin/14-3-3σ	Cell cycle checkpoint protein	Stratifin promotes ΔNp63α nuclear export into the cytoplasm, and then facilitates degradation of ΔNp63α by RACK1	[191]

The WW domain containing E3 ubiquitin protein ligase 1 (WWP1) is a member of HECT type E3 family. WWP1 physically associates with both TAp63α and ΔNp63α via PY/WW motif interaction, leading to ubiquitination and degradation of TAp63α and ΔNp63α proteins [188]. Phosphorylation of the conserved threonine (T538) close to the PxY motif of p63α is critical for Itch-p63α binding [186], but is not required for WWP1 mediated p63α degradation, as T538A substitution fails to affect WWP1-mediated proteasomal degradation [189]. Pin1 directly binds to p63α, but not the γ isotypes, and prevents p63α degradation by E3 ligase WWP1 [189]. A recent study revealed that WWP1 contributes to metformin-induced ΔNp63α degradation in HNSCC FaDu cells [190].

RACK1 is a WD-40 repeat-containing scaffold protein binding to p63α [181, 191]. Upon DNA damage, ΔNp63α protein is exported from the nucleus and into the cytoplasm, where it is recognized by RACK1. RACK1 binds the C-terminal SAM of p63 via its two WD40 repeats in C-terminus and promotes p63 proteasomal degradation [181, 191]. RACK1-dependent degradation of the ΔNp63α protein can be inhibited by Stxbp4 [181]. Upon DNA damage, stratifin physically interacts with ΔNp63α at its SAM motif and facilitates its nuclear export into the cytoplasm, and then facilitates degradation of ΔNp63α, mediated by RACK1 [191].

## Conclusions and perspectives

Squamous cell carcinoma remains a great challenge to human health due to its highly aggressive and limited therapeutic options. SCC from various organs is highly dependent on ΔNp63α, which is recently highlighted for its pioneer activity and ability to establishing active enhancers through interaction with epigenetic regulators. The existence of p63 in chromatin-remodeling complexes provides an opportunity to target p63-positive SCCs via epigenetic therapy. However, many of the interaction partners of p63 in keratinocytes, including KMT2D and SWI/SNF components, are frequently mutated in SCC, implying that the p63-mediated differentiation transcriptional program is disturbed during SCC. It remains unclear how ΔNp63α cooperates with epigenetic regulators to activate enhancer activity of oncogenes in SCC. Additional efforts are necessary to fully understand the complexity of the interactions between ΔNp63α and epigenetic regulators in the control of SCC development. Characterization of ΔNp63α-binding partners in SCCs may enable the development novel strategies to indirectly interfere with ΔNp63α activity. Disruption of the interaction between ΔNp63α and diverse coactivators in SCC cells will broadly affect ΔNp63α-dependent transcription and may provide a novel therapeutic approach for SCC. ΔNp63α expression is sufficient to establish a squamous subtype-specific SE to drive oncogene expression in SCC. Thus, another area worthy of attention is the mechanisms underlying phase separation of ΔNp63α-dependent SE in SCC cells. It has been shown that ΔNp63α interacts with the c-Rel subunit of nuclear factor-kappa B (NF-κB) in keratinocytes and HNSCC cells [192]. Given the central role of NF-κB in the immune system and cancer development, the cooperation between ΔNp63α and c-Rel may influence the tumor microenvironment [193]. Immunosuppressive tumor microenvironment facilitates SCC development, progression, and therapeutic resistance [194, 195]. A substantial proportion of SCC patients do not respond to immune checkpoint blockade therapy, including PD-1/PD-L1 mAb treatment [2, 196, 197]. To overcome barriers to effective immunotherapy, we believe that clarification is needed concerning how ΔNp63α cooperates with NF-κB and other immune-regulatory factors to alter the immune environment of SCC.

## Abbreviations

SCC: Squamous cell carcinoma
HNSCC: Head and neck squamous cell carcinoma
TF: Transcription factor
ECM: Extracellular matrix
iPSCs: Induced pluripotent stem cells
RA: Retinoic acid
BMP4: Bone morphogenetic protein 4
hESCs: Human embryonic stem cells
KLF4: Kruppel-like factor 4
eRNAs: Enhancer RNAs
EpSCs: Epidermal stem cells
ChIP: Chromatin immunoprecipitation
PRC: Polycomb repressive complex 1
PC: Pancreatic cancer
PDA: Pancreatic ductal adenocarcinoma
SE: Super enhancer
COMPASS: Complex of proteins associated with set1-like complex
CBP: CAMP response element-binding protein
GCN5: General control nonrepressed protein 5
SWI/SNF: Switch/sucrose nonfermentable
EDC: Epidermal differentiation complex
Satb1: Specialized adenine and thymine-rich binding protein 1
Dnmt3a: DNA methyltransferase 3A
5-hmC: 5-Hydroxymethylcytosine
DNMT: DNA methyltransferase
LSH: Lymphoid-specific helicase
METTL3: Methyltransferase-like 3
NUP62: Nucleoporin 62
APC/C: Anaphase-promoting complex/cyclosome
RACK1: Receptor for protein kinase C1
WWP1: WW domain containing E3 ubiquitin protein ligase 1
